# Distribution and Expression of Programmed Death Ligand -1 (PD-L1) in Non-Small Cell Carcinomas of the Lung in a Tertiary Care Centre in South India

**DOI:** 10.5146/tjpath.2021.01525

**Published:** 2021-05-15

**Authors:** Sheba S K Jacob

**Affiliations:** Apollo Hospitals, Department of Pathology, Chennai, India

**Keywords:** PD-L1, NSCLC, Non-small cell lung carcinoma, Pembrolizumab, Immune checkpoint inhibitors

## Abstract

***Objective:*** Non-small cell lung carcinomas often present at an advanced stage with a grim prognosis. Immune checkpoint (ICP) inhibitors have drastically changed the scenario, and the response to ICP inhibitors is determined by analyzing the expression of PD-L1 by immunohistochemistry. PD-L1 immunohistochemistry helps in optimizing the treatment, and avoiding unnecessary exposure of patients to the toxic effects of the drugs that are ineffective and expensive in non-expressing tumors. This study was conducted to assess the prevalence of the expression of PD-L1 in non-small cell carcinomas of the lung diagnosed at our institution, which is a tertiary care center in South India.

***Material and Method:*** The PD-L1 immunohistochemistry of 77 cases of non-small cell carcinomas of the lung diagnosed over a period of two years were reviewed and analyzed (2018-2020). All tissues were fixed in 10% neutral buffered formalin and processed by standard methods, and the Ventana SP263 clone was used.

***Results:*** Seventy-seven cases of non-small cell lung carcinomas were reviewed and studied for (PD-L1) expression. 35/77 (45%) of the cases had PD-L1 expression (≥ 1%) and 14 (18 %) had high (PD-L1) expression. Also there was a male preponderance of 2.3:1. High PD-L1 expression was seen mostly in patients above 60 years of age and was usually associated with high tumor grade.

***Conclusion:*** It is important to assess PD-L1 expression in non-small cell carcinomas of patients especially with higher tumor grade and older age groups that they may benefit from immune checkpoint inhibitor therapy.

## INTRODUCTION

Tumor cells express programmed death ligand -1 (PD-L1) to produce an immunosuppressive milieu in the local tissue microenvironment for evading immunity as one of the modes of survival and this is associated with an aggressive course (1–3). PD-L1 (CD274), a type 1 transmembrane protein, plays a major role in suppressing the adaptive arm of the immune system. The binding of PD-L1 to the inhibitory checkpoint molecule PD-L1 transmits an inhibitory signal, reducing the proliferation of antigen-specific T-cells in lymph nodes, thereby preventing an immune response to the cell. Qualitative detection of the PD-L1 protein by immunohistochemistry in formalin-fixed, paraffin-embedded non-small cell lung carcinomas (NSCLC) helps in identifying patients suitable for treatment with immune checkpoint inhibitors which are associated with enhanced survival (4). Hence, immune checkpoint inhibitors have changed the way of treatment of lung carcinoma and have an impact on the prognosis (5). This study was done to assess the prevalence of the expression of PD-L1 in non small cell carcinomas of the lung diagnosed at our hospital, which is a tertiary care center in South India, over a period of two years between 2018 and 2020.

Lung carcinoma is one of the leading causes of cancer related mortality throughout the world (6). About 80% of these are non-small cell lung carcinomas (7), often presenting at an advanced stage and the prognosis is often grim (8). Immune checkpoint (ICP) inhibitors have drastically changed the scenario, especially when there is a good expression of PD-L1. The response to ICP inhibitors has to be determined by analyzing the expression of PD-L1 by immunohistochemistry as the non-expressing tumors are resistant to this therapy (9). So PD-L1 immunohistochemistry helps in optimizing the treatment, avoiding unnecessary exposure of patients to the toxic effects of the drugs that are ineffective and expensive in non-expressing tumors, and is also a prognostic biomarker in non-small cell carcinomas.

## MATERIAL and METHODS

The PD-L1immunohistochemistry of 77 cases of non-small cell carcinomas of the lung diagnosed over a period of two years were reviewed and analyzed (2018-2020). All biopsies/ specimens were fixed in 10% neutral buffered formalin and processed by standard methods and stained with the Hematoxylin and Eosin stain. PD-L1 staining by immunohistochemistry was carried out on 4 μm sections, using the Ventana SP263 clone (10), OptiView Amplification Kit, and OptiView DAB IHC Detection Kit on Ventana Benchmark XT equipment. Hematoxylin was used as a counterstain. The tumor proportion score (TPS) of PD-L1 was calculated as the percentage of viable tumor cells with membrane positivity. Tumors with ≥50% TPS were considered to have high expression of PD-L1. Tumors with ≥ 1 to <50% TPS were considered to have low expression of PD-L1. Tumours with no or <1% TPS were considered negative.

All cases of non-small cell carcinomas of the lung primary/metastatic, received in the department of pathology of Apollo hospitals during the period 2018-2020 with PD-L1 immunohistochemistry were reviewed. Exclusion criteria included non-small cell carcinomas of the lung without PD-L1 immunohistochemistry or PD-L1immunohistochemistry in carcinomas of other sites.

### Statistical Analysis

The prevalence of PD-L1 TPS ≥ 50%, TPS ≥ 1%, and TPS < 1% was summarized using counts and percentages. As there were no a priori hypotheses, no P values were determined.

The study was approved by the Institutional Ethics Committee (Ref No: AMH-C-S-023/08-20; Date: 01 September 2020).

## RESULTS

The PD-L1 staining by immunohistochemistry of 77 cases of non-small cell lung carcinomas diagnosed during the period of 2018-20 were evaluated retrospectively. Of these, 52 were male and 25 were female and the age range was 35-87 years. The median age was 66 years. Most of the samples were from the lung, 54 were biopsies, and 3 were resections of primary lung carcinomas. Metastatic non-small cell lung carcinomas from 11 pleural biopsies, 2 cervical lymph nodes, 1 axillary lymph node and 1 sample from the D12 vertebra were also studied. Five cell blocks from pleural fluid were also studied. Of the primary lung carcinomas, 47 were adenocarcinomas, 6 were squamous cell carcinomas, one was adenocarcinoma in situ and 3 were adenosquamous carcinomas. Five cell blocks from pleural effusion had malignant cells from lung non-small cell carcinoma and were analyzed for the expression of PD-L1. 14/15 of the metastatic carcinomas were adenocarcinomas and one was a sarcomatoid carcinoma.

PD-L1 positivity was noted in 35/77 cases with 14 of them showed high PD-L1 expression (≥50% TPS). The male to female ratio was 2.3:1 and the age range was 42 to 87 years and a median age of 66 years. High PD-L1 expression was noted in the age range of 57-80 years and most were above 60 years of age (Table 1). Very low PD-L1 expression (<1%) which is considered negative was noted in 12 cases. PDL-1 was not expressed in 30 cases and of these 22 were male and 8 were female. 47% and 46% of the males and females studied showed PD-L1 expression, respectively.

**Table 1:** Age and PD-L1 expression

**Age range**	**PD-L1 expression**		
	**Negative**	**Low**	**High**
**31-40**	1		
**41-50**	5	5	
**51-60**	10	4	3
**61-70**	16	8	6
**71-80**	8	2	5
**81-90**	2	2	

**Figure 1:**
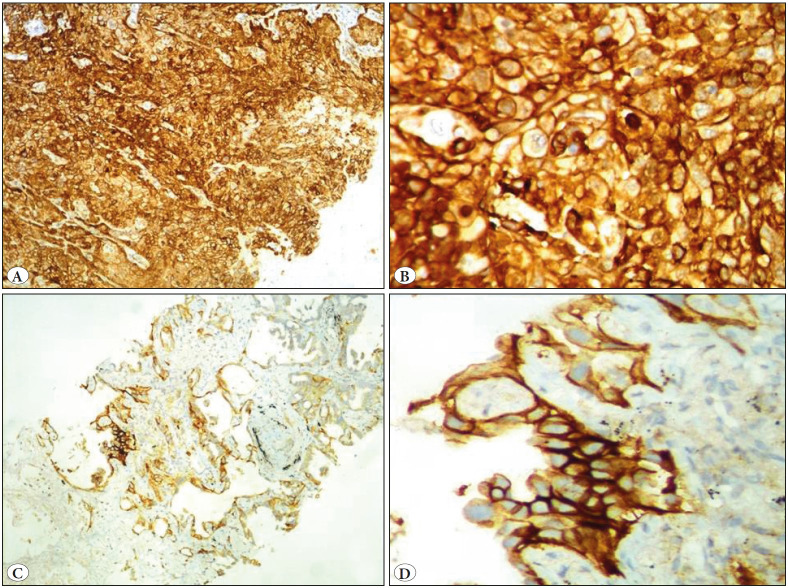
**A-B)** High PD-L1 expression in a squamous cell carcinoma (IHC; A: x40 & B: x400). **C-D)** Low PD-L1 expression in an adenocarcinoma (IHC; C: x40 & D: x400).

Interestingly 4/6 (60%) grade 2/3 squamous cell carcinomas were negative. One grade 3 tumor showed low positivity and another grade 2 tumor showed high positivity (Figure 1).

Of the primary adenocarcinomas of the lung tested for PD-L1, 43% showed positivity (Figure 1). Eleven (65 %) of the grade 3 carcinomas showed ≥ 1% TPS and six were negative. 18 (67%) of the grade 2 adenocarcinomas were negative. Nine cases (33 %) were positive. All the three grade 1 adenocarcinomas were negative (Table 2).

**Table 2:** Tumor grade and PD-L1 expression

**PD-L1 status (TPS)**	**AC** **G1**	**AC** **G2**	**AC** **G3**	**SCC** **G1**	**SCC** **G2**	**SCC** **G3**	**AS** **G3**	**AIS**	**MC**	**PLEURAL FLUID** **-Ma C**
**Neg**	3	18	6		1	3			9	2
**Low**		8	2			1	2	1	5	2
**High**		1	9		1		1		1	1

**NEG:** Negative, **TPS:** Tumor proportion score, **G:** Grade, **AC :** Adenocarcinoma, **SCC:** Squamous cell carcinoma, **AS:** Adenosquamous carcinoma, **AIS:** Adenocarcinoma in situ, **MC:** Metastatic carcinoma, **MaC:** Malignant cells.

All the three adenosquamous carcinomas were positive with two showing low positivity. One adenocarcinoma in situ had low positivity.

Seven pleural and one each of the metastatic adenocarcinoma to the axilla and vertebra cases were negative or <1%TPS. Two cervical lymph node metastases were positive and four of the metastatic carcinoma in the pleura had low expression.

## DISCUSSION

This study was conducted at a tertiary care hospital in Chennai, Tamilnadu, South India, and is the first of its kind. The patients are not only from the state of Tamilnadu and South India but are also from the northeastern part of India. 77 cases of non-small cell lung carcinomas diagnosed over a period of two years (2018-2020) were reviewed and studied for PD-L1 expression. 45% of the non-small cell lung carcinomas had PDL-1 expression (≥ 1%) and 18% had high PD-L1 expression. PD-L1 expression is associated with a poor prognosis (11–13) unless treated with ICP inhibitors which significantly improve the survival. Patients with high PD-L1 expression are treated with first-line treatment, pembrolizumab. Those with low PD-L1 expression are treated with second-line drugs, pembrolizumab, and they may be associated with enhanced survival on treatment with nivolumab. Durvalumab can be used in post chemoradiotherapy patients with low PD-L1 expression. In patients with advanced NSCLC and high PD-L1 expression, pembrolizumab was associated with a longer progression-free survival and with fewer adverse events than with platinum-based chemotherapy (14). 55% had <1% or no expression of PD-L1, compared to 48% in an international multicenter study (15).

The prevalence of (PD-L1) of TPS ≥ 1% in a large multicenter study from different continents was 52% in Europe, 53% in Asia-Pacific, 47% in the Americas, and 55% in other countries but ours was 45% which is comparable to the Americas, slightly lower than European countries but higher than in a Brazilian study which had 24 % (8) and an Indian study (34%) (16). However, only stage IIIB/IV cases of NSCLC were included in the large multicenter study (15). Only 24% of the non-small cell lung carcinomas expressed PD-L1 in a German study (17). 34.5% of the non-small cell lung carcinomas had PD-L1 expression in a Japanese study of only surgically resected specimens (18). These studies from different countries/continents indicate that the percentage of expression of (PD-L1) is variable in different populations but this may also be due to the different clones used in these studies or interpersonal bias. Also the PD-L1expression in a small biopsy of the tumour may not be representative of the entire lesion (19).

High (PD-L1) expression was seen mostly in patients above 60 years of age in our study (Table 1) but a study from Australia showed that a younger population and high tumor grade resulted in a high (PD-L1) expression (3). High PD-L1 was associated with high tumor grade which was comparable to our study (3,13,20,21). The prevalence of high PD-L1 expression (TPS ≥ 50%) was 22% in Europe, 22% in Asia-Pacific, 21% in the Americas, and 24% in other countries (15), comparable to 18% in our study but this is slightly higher than the Brazilian study (16.5%). 32% of the non-small cell carcinomas showed high PD-L1 expression in a study from west Australia (22). The expression is variable in different Chinese studies with one of them showing the highest expression of PD-L1 (66.8%) (23) but in another Chinese study the expression was 33.7 % with 10.8% showing high positivity (24). Also there was a male preponderance (69%) comparable to our study which was 70% (16). 47% and 46% of the males and females studied showed PD-L1 expression respectively compared to 52% each in the large multicenter study (15). The PD-L1 positivity in an Indian study was 35.5% and 28% in males and females respectively which is lower than in our study (16).

About 42 and 33 percent of the adeno and squamous cell carcinomas showed positivity in our study compared to 57% and 51% in the international study, respectively (15). There was a higher expression of PD-L1 in squamous cell carcinomas (60%) compared to adenocarcinomas (22%) in a Japanese study (2). The tumor proportion score was also higher in squamous cell carcinoma in a study from Western Australia (22). However, the percentage of expression did not vary with the tumor type in other studies.

A higher expression of PD-L1 was noted in high grade adenocarcinomas in the Japanese study comparable to our study. However, no such correlation was noted for squamous cell carcinomas (2). Interestingly, PD-L1 expression on tumor cells was associated with improved overall survival in pulmonary squamous cell carcinomas with adjuvant therapy in spite of increased tumor size and positive lymph node status (17).

Only 44% of the metastases showed positivity compared to 53% in the large multicenter study. 20% of the cases with pleural effusion showed positivity in another study while three of the five cases of pleural effusion showed positivity in our study (16).

On evaluation, 33 and 65% of the moderately and poorly differentiated carcinomas showed positivity, respectively, compared to 13.79%, and 36.11% in an Indian study. All adenosquamous carcinomas were high grade and showed positivity in our study while only 40.9% of adenosquamous carcinomas showed positivity in that study (16). A study from China shows promising results with immune checkpoint inhibitors in cases with adenosquamous carcinomas (25).

Another interesting finding is the observation of high PD-L1 expression and lack of treatable driver mutation raises the potential of checkpoint immunotherapy for the rare type of lung tumor - lymphoepithelioma-like carcinoma (26).

Some studies have pointed out that the PD-L1 study may be repeated if there is a shift in the stage or if we are able to get a larger biopsy, as the biopsy taken at a lower stage and a smaller sample may not have the same expression at a higher stage or in other foci (19,27).

There was no significant correlation between PD-L1 expression and molecular or genetic abnormalities, or other parameters including age, gender, stage, and smoking status (22).

In conclusion, PD-L1 expression has a male preponderance and high PD-L1 was associated with higher tumour grade and older age group >60 years. It is important to assess the PD-L1 expression in non-small cell carcinomas of patients especially with higher tumor grade and in the older age group so that they may benefit from immune checkpoint inhibitor therapy. The expression of PD-L1 in non-small cell carcinomas is only slightly variable in different populations.

## Conflict OF INTEREST

The authors declare no conflict of interest.

## FUNDING

This study did not receive any specific grant from funding agencies in the public, commercial, or not-for-profit sectors.
